# Salinity Stress Responses and Adaptation Mechanisms of *Zygophyllum propinquum*: A Comprehensive Study on Growth, Water Relations, Ion Balance, Photosynthesis, and Antioxidant Defense

**DOI:** 10.3390/plants13233332

**Published:** 2024-11-28

**Authors:** Bilquees Gul, Sumaira Manzoor, Aysha Rasheed, Abdul Hameed, Muhammad Zaheer Ahmed, Hans-Werner Koyro

**Affiliations:** 1 Dr. Muhammad Ajmal Khan Institute of Sustainable Halophyte Utilization, University of Karachi, Karachi 75270, Pakistan; halophyte_aysha@yahoo.com (A.R.); ahameed@uok.edu.pk (A.H.); mzahmed@uok.edu.pk (M.Z.A.); 2 Department of Botany, Government Degree D.J. Science College, Karachi 74400, Pakistan; smrmnzr@gmail.com; 3 Interdisziplinäres Forschungszentrum (IFZ), Institut für Pflanzenökologie, Heinrich-Buff-Ring 26, 35392 Gießen, Germany; hans-werner.koyro@bot2.bio.uni-giessen.de

**Keywords:** halophyte, salinity, ion content, chlorophyll fluorescence, antioxidant defense system

## Abstract

*Zygophyllum propinquum* (Decne.) is a leaf succulent C_4_ perennial found in arid saline areas of southern Pakistan and neighboring countries, where it is utilized as herbal medicine. This study investigated how growth, water relations, ion content, chlorophyll fluorescence, and antioxidant system of *Z. propinquum* change as salinity levels increase (0, 150, 300, 600, and 900 mM NaCl). Salinity increments inhibited total plant fresh weight, whereas dry weight remained constant at moderate salinity and decreased at high salinity. Leaf area, succulence, and relative water content decreased as salinity increased. Similarly, the sap osmotic potential of both roots and shoots declined as NaCl concentrations increased. Except for a transitory increase in roots at 300 mM NaCl, sodium concentrations in roots and shoots increased constitutively to more than five times higher under saline conditions than in non-saline controls. Root potassium increased briefly at 300 mM NaCl but did not respond to NaCl treatments in the leaf. Photosynthetic pigments increased with 300 and 600 mM NaCl compared to non-saline treatments, although carotenoids appeared unaffected by NaCl treatments. Except for very high NaCl concentration (900 mM), salinity showed no significant effect on the maximum efficiency of photosystem II photochemistry (Fv/Fm). Light response curves demonstrated reduced absolute (ETR^*^) and maximum electron transport rates (ETR_max_) for the 600 and 900 mM NaCl treatments. The alpha (α), which indicates the maximum yield of photosynthesis, decreased with increasing NaCl concentrations, reaching its lowest at 900 mM NaCl. Non-photochemical quenching (NPQ) values were significantly higher under 150 and 300 mM NaCl treatments than under non-saline and higher NaCl treatments. Electrolyte leakage, malondialdehyde (MDA), and hydrogen peroxide (H_2_O_2_) peaked only at 900 mM NaCl. Superoxide dismutase and glutathione reductase activities and glutathione content in both roots and shoots increased progressively with increasing salinity. Hence, growth reduction under low to moderate (150–600 mM NaCl) salinity appeared to be an induced response, while high (900 mM NaCl) salinity was injurious.

## 1. Introduction

Soil salinity is one of the important environmental stresses in arid and semi-arid climates, which adversely impacts crop yield and plant growth, ultimately affecting the ecosystem’s primary productivity [1,2,3]. It has an impact on plants at all lifecycle stages, including germination [4,5,6], growth and development [1] distribution patterns [7,8], and biological interactions [8,9]. Plants exhibit reduced growth when exposed to salt due to osmotic constraints, ion toxicity, nutritional imbalance, reduced photosynthesis, and oxidative damage [10,11,12,13,14,15,16]. However, halophytes have evolved adaptations to resist high soil/water salinity, which is fatal to most other plants [17,18]. Xero-halophytes can endure both drought and salinity, giving them a high potential for increasing salt desert productivity in various ways [19,20]. Many xero-halophytes, such as *Zygophyllum xanthoxylum* [21], *Haloxylon ammodendron* [22] *Kosteletzkya virginica* [23], *Atriplex halimus* [24], and *Nitraria tangutorum* [25], have been identified as potential candidates for growth and establishment in saline environments. However, a lack of understanding of xero-halophytes’ physiological and biochemical responses to abiotic stresses limits their proper utilization [26].

With the concurrent accumulation of suitable organic solutes, osmotic adjustment and ion compartmentalization in cell vacuoles are two closely related mechanisms that allow halophytes to adapt to the direct impacts (osmotic and ionic stressors) of high salinity [27]. One important characteristic of salinity tolerance in plants is Na^+^ partitioning between the biomass of the roots and the shoots [28]. *Atriplex halimus* [29,30], *Sesuvium portulacastrum* [31], and *Z. xanthoxylum* [32,33] are the examples of xero-halophytes that accumulate Na^+^ in their shoots. This helps these plants withstand water stress caused by osmotic imbalance under high soil salinity levels. *Atriplex canescens* uses Na^+^ uptake directly for osmotic adjustment [34]. Moreover, dumping salts into old foliage may also help deal with salinity [27,28,35]. Active compartmentalization of excess Na^+^ and Cl^−^ in vacuoles minimizes salt toxicity and helps osmoregulation. Numerous studies have shown that Na^+^ improves photosynthesis and water status and bolsters antioxidant activities in many halophytes in arid saline conditions [32,33,36].

One potential method for measuring a plant’s performance to stress conditions is chlorophyll fluorescence [37,38]. Because the parameters often assessed are directly related to photosystem II (PSII) functioning, they can be used to assess plant resistance under adverse environmental conditions [39]. Although plants’ photosynthetic apparatus is negatively impacted by salinity, many salt-tolerant plants do not exhibit any symptoms of photo-inhibition [40]. As a result of a reduction in the photosynthetic electron transport chain caused at high salt concentrations, alternate mechanisms to prevent photo-inhibition of PSII may be induced [40,41,42]. Plants use the xanthophyll cycle as an effective defense mechanism to dissipate excess energy in PSII [43,44,45]. In contrast, the water-water cycle shields PSI by scavenging reactive oxygen species ROS) [46,47,48,49]. However, not all plants have the same levels of efficacy in these defense mechanisms for photosynthesis [50,51,52]. Excess light energy is primarily dissipated by chlorophyll fluorescence and non-photochemical quenching [37,44]. The regulation of non-photochemical quenching (NPQ) and fluorescence verifies the extent of plant responses to salinity and the efficiency of PSII [53,54,55]. Plants have adapted to improve water-use efficiency through CO_2_ accumulation and increased productivity through the C_4_ mode of photosynthesis [11,56,57]. C_4_ plants are better able to tolerate salinity stress than C_3_ plants, and can better occupy and establish saline areas [58]. As a result, C4 plants can survive harsh environmental conditions [58,59].

One of the major changes in plant biochemistry caused by salinity is oxidative stress due to excessive reactive oxygen species (ROS) production [16,55,60]. Increased ROS levels can inflict oxidative stress, characterized by oxidative damages to numerous cell components, such as nucleic acids, membrane lipids, and proteins [61,62,63]. The primary negative impact of oxidative stress is membrane lipid breakdown or lipid peroxidation, which may result in the generation of malondialdehyde (MDA) [55,64,65], which therefore serves as an indicator of oxidative stress induced by salt stress [66]. Plants have evolved complex antioxidant defense systems with enzymatic and non-enzymatic processes to combat excess ROS generation [67]. Antioxidative enzymes include superoxide dismutases (SOD), catalases (CAT), guaiacol peroxidase (POD), ascorbate peroxidase (APX), and glutathione reductase (GR) [16,61,67]. SOD is the first line of defense against oxidative stress and converts superoxide (O2-) into hydrogen peroxide (H_2_O_2_). CAT and other peroxidases (POD) detoxify H_2_O_2_ [67]. Ascorbate (AsA) and glutathione (GSH) are common antioxidant substrates that quench ROS directly or indirectly through enzymatic activity. Antioxidant defense efficiency and salt tolerance are frequently positively correlated [68,69]. However, this information is missing for the majority of subtropical xero-halophytes.

*Zygophyllum propinquum* is a leaf succulent xero-halophyte that grows in arid saline areas of the Saharo-Sindian region [70,71]. It is used locally as a herbal treatment for diabetes, asthma, gout, rheumatism, antihelminthic, and hypertension [70,72]. Numerous phytochemical investigations have demonstrated its antihelminthic, antipyretic, anesthetic, and diuretic effects [73,74,75]. The majority of research has focused on the chemical analysis of this plant [76,77,78], but no examination has been conducted to understand the salt tolerance mechanism during the growth of this medicinally significant xero-halophyte. This study aimed to assess the effects of different NaCl concentrations on growth, water-related parameters, ion contents, chlorophyll fluorescence, and antioxidant processes of *Z. propinquum*.

## 2. Materials and Methods

### 2.1. Seed Collection Site, Growth Conditions, and NaCl Treatment

Seeds of *Zygophyllum propinquum* were collected from a large population near the Hub Dam in Karachi. The seed collection location was a dry salt flat with gravely sandy soil and a warm subtropical monsoon climate [79]. Inflorescence were manually cleaned to extract seeds and briefly stored at room temperature before the experiment. Seeds were planted in plastic tubs in soil culture (1:1; sandy loam soil: black farm manure) in a green net house and irrigated daily with tap water for 8 weeks. Seedlings were transplanted into pots (12 cm Diameter × 15 cm Depth) with quartz sandy soil. Plants were sub-irrigated through holes at the bottom of pots by placing them in plastic trays (32 cm Diameter × 6 Depth) containing 2 L of half-strength Hoagland’s nutritional solution [80]. At noon (1 pm), a portable weather station recorded the following environmental conditions: relative humidity of 55–65%, light intensity of 250–350 μmoles m^−2^ s^−1^, photoperiod of 12–15 h per day from sunshine, and temperature of 32–38 °C. Salt treatments were applied at concentrations of 0, 150, 300, 600, and 900 mM NaCl. Based on these levels, salt stress was classified as low (150 mM), moderate (300 mM), high (600 mM), and very high (900 mM), as shown in Table 1, to clearly distinguish the degrees of salt tolerance in response to NaCl treatments. To minimize osmotic stress, salt treatments were increased progressively, at increments of 50 mM NaCl every 12 h, starting eight weeks after transplantation. Treatment concentrations in the trays were measured using a hand-held refractometer (Hand Refractometer, S-10E, ATAGO, Tokyo, Japan) and replenished twice daily with tap water to account for evaporative loss. Furthermore, the plastic pots were flushed with the appropriate irrigation solutions daily and allowed to drip from below to avoid salinity buildup. The treatment medium (NaCl + nutritional solution) was renewed at 5-day intervals. Plants were harvested 15 days after reaching the final salt concentrations.

### 2.2. Growth Parameters, Leaf Area, and Succulence

Plants were selected and rinsed with the appropriate treatment solution. This was followed by rapid rinsing with tap water and drying with blotting paper. Plants length and fresh weight (FW) were measured immediately. Dry weight (DW) was determined by drying the plant samples at 60 °C for 72 h or until they reached a consistent weight. The projected leaf area per plant was calculated using ImageJ software ImageJ (version 1.46) and scaled pictures of the leaves on a white backdrop. Succulence, defined as the water content per unit dry weight, was computed using the following formula:

Succulence (g H_2_O g^−1^ DW) = (FW − DW)/DW

All physiological and biochemical analyses in this study were performed on mature, fully expanded leaves from the 2nd and 3rd nodes.

### 2.3. Relative Water Content (RWC)

Leaf fresh weight (FW) was measured immediately after harvest, and the leaves were immersed in distilled water at room temperature (28 to 30 degrees Celsius) for 24 h. The leaves were then gently blotted dry to determine the turgid weight (TW) and oven-dried at 60 °C before recording the dry weight (DW). The formula used to calculate the RWC is as follows:

RWC (%) = (FW − DW)/(TW − DW) × 100

### 2.4. Water Relations

Leaf and root osmolality was determined by expressed sap according to [15] using a vapor pressure osmometer (VAPRO-5520; Wescor Inc., Logan, UT, USA). Osmolality readings were converted to osmotic potential (ΨS) in MPa by using Vant Hoff’s equation ΨS = −cRT, where c is the number of moles of solute, R = 0.008314 J mol^−1^ K^−1^ (gas constant), and T = 298.8 K [81]. Similarly, the osmolality of the irrigation solutions used for irrigating the plants was also determined. We also calculated the percent osmotic contribution of Na^+^ to leaf and root ΨS [81].

### 2.5. Cation Contents

Oven-dried plant materials (roots and leaves) were converted to ash in a muffle furnace at 550 C for five hours. The ash samples were then digested in 1 mL of 20% HCl at 60 °C on a hot plate for 15 min. The samples were allowed to cool to room temperature, dissolved in distilled water, and passed through Whatman No. 42 filter paper. levels of Na^+^ and K^+^ were then measured using a flame photometer (Gentaur Intech model I-66, Brussels, Belgium). They were expressed in mmol g^−1^ tissue DW (content basis) and mmol L^−1^ tissue sap (concentration basis) and based on whole tissue-specific distribution. Ion data were used to calculate the Na/K ratio at both tissue levels. Moreover, the estimated selective absorption ratio of K^+^ vs. Na^+^ from the medium (SA) was calculated using the following formula

[K^+^/(K^+^ + Na^+^)_root_]/[K^+^/(K^+^ + Na^+^)_Medium_]

### 2.6. Determination of Photosynthetic Pigments

The chlorophyll extract from freshly collected leaves was extracted with 100% ethanol in tightly capped glass test tubes and stored in the dark at 4 °C for 3 days. The chlorophyll extract was collected in another set of test tubes and replaced with a similar volume of ethanol. This procedure was repeated for 3–4 days, followed by centrifugation at 4 °C until the leaves were almost completely discolored. Pigment estimation was carried out using a spectrophotometer (UV-vis spectrophotometer DU-730, Beckman-Coulter, CA, USA).

### 2.7. Chlorophyll Fluorescence Measurements

Mature leaves of plants after 15 days of NaCl treatment were utilized to measure chlorophyll fluorescence (Chl a) light response curves (LRCs) in steady state using a PAM fluorometer 2500 (Heinz Walz GmbH, Effeltrich, Germany). Dark-adapted leaves were utilized to detect minimal fluorescence (Fo), and maximal fluorescence (Fm) was approximated to obtain the maximum photochemical quantum yield of PSII. After the dark phase, the leaves were exposed to intense light (1300 µmol photon m^−2^ s^−1^) for 20 min. LRCs were then evaluated at decreasing irradiance levels. The measured values of alpha (α)—the early slope of the light curve, ETRmax—the maximum electron transfer values, Ik—the junction of the horizontal line ETRmax, and Fv/Fm × ETR factor/2 were recorded from the instrument. [82] equation was used to calculate Stern-Volmer type non-photochemical quenching (NPQ), whereas [53] equation was used to calculate the effective quantum yield of PS II (YII) as YII = Fm′; − Fs/Fm′. The coefficient of photochemical fluorescence quenching (qP) was calculated using the formula qP = (Fm′ − Fs)/(Fm′ − Fo′) [83,84]. Ref. [85] reported quantum yields for the controlled energy dissipation (YNPQ) and non-regulated quenching (YNO) of PS II, as described by [86]. The absolute electron transfer rate (ETR*) using leaf absorbance measured with an integrating sphere (Li 1800-12, LiCor Inc., Lincoln, NE, USA).

### 2.8. Electrolyte Leakage

Electrolyte leakage (%) was determined by immersing leaves in de-ionized water, and the electrical conductivity (EC) was measured before and after autoclaving for 20 min according to [87]. The following formula was used:

E_1_/E_2_ × 100

E_1_ and E_2_ are the electrical conductivity values taken before and after autoclaving the material.

### 2.9. Oxidative Stress Markers

Freshly ground plant materials (root and leaf) were homogenized in 5 mL of 3% Trichloroacetic acid (TCA), followed by centrifugation at 12,000× *g* for 15 min at 4 °C to prepare extracts. Hydrogen peroxide (H_2_O_2_) content was estimated according to the KI reagent assay of [88]. The extent of lipid peroxidation was estimated by determining the contents of malondialdehyde (MDA). Malondialdehyde (MDA) contents were determined by using 2-thiobarbituric acid test, according to [89].

### 2.10. Antioxidant Enzyme Activities

The extraction of antioxidant enzymes was performed following the protocol described by [90]. Superoxide dismutase (SOD, EC 1.15.1.1) activity was measured at 560 nm by [91]. Catalase (CAT, EC 1.11.1.6) activity was assayed by [92] method at 240 nm. Guaiacol peroxidase (GPX, EC 1.11.17) activity was measured at 470 nm by [93] protocol. Ascorbate peroxidase (APX, EC 1.11.1.11) activity was examined according to [94] at 290 nm. Glutathione reductase (GR, EC 1.6.4.2) activity was quantified at 340 nm by method [95]. Protein content of enzyme extracts was determined according to [96], and enzyme activities were expressed as units per milligram of the protein.

### 2.11. Antioxidant Substances

Contents of reduced ascorbic acid (AsA) were determined in TCA extracts according to the method of [97]. Glutathione (GSH) contents were quantified in TCA extracts by following the 5,5-dithiobis-(2-nitrobenzoic acid) method of [98].

### 2.12. Statistical Analyses

For statistical analysis of data, Ref. [99] was used. One-way analysis of variance (ANOVA) was performed to test whether the experimental factor (NaCl concentration) affected various plant parameters significantly. Post-hoc Bonferroni analysis (*p* < 0.05) was carried out to indicate significant differences among individual treatment means.

## 3. Results

### 3.1. Effects of Salinity on Plant Growth

**Figure 1:**
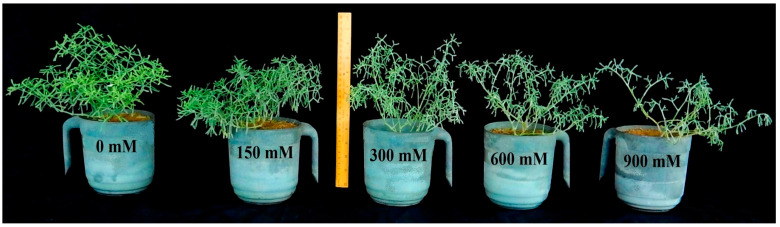
Comparison of *Zygophyllum propinquum* plants grown under different (mM) NaCl treatments for 15 days under a green net house.

**Figure 2:**
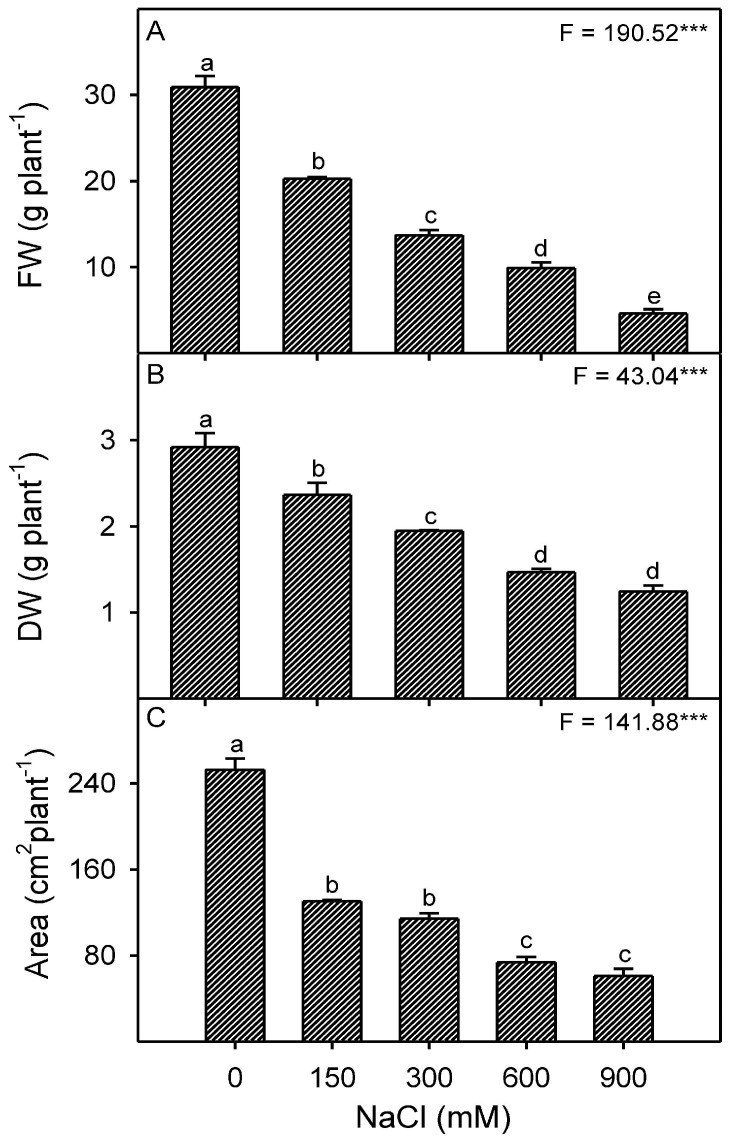
Morphological changes of *Zygophyllum propinquum* leaves in response to different NaCl treatments (0, 150, 300, 600, and 900 mM) (**A**) fresh weight (FW g^−1^ plants); (**B**) dry weight (DW g^−1^ plants); (**C**) leaf area (cm_2_ plant^−1^). Bars represent the mean ± standard error (n = 3). Bars with different letters are significantly different from each other (*p* < 0.05; post-hoc test). *F*-values based on one-way ANOVA for the effect of salinity are given. Where, *** = *p* < 0.001.

Plant growth was optimal in the non-saline control condition but reduced as salinity increased (Figure 1). NaCl treatments did not impact root length, but shoot length decreased significantly (*p* < 0.001) at 600 and 900 mM NaCl compared to the non-saline control (Table 1). Under saline conditions, root fresh weight (FW) decreased to nearly 50% of the control, with little variation between NaCl treatments. However, shoot FW decreased (*p* < 0.001) steadily with increasing NaCl treatments. However, root and shoot dry weights (DW) remained nearly constant, with a little reduction at high NaCl concentrations. As NaCl treatments increased, leaf biomass and area decreased significantly (*p* < 0.001) compared to the non-saline control (Figure 2A–C).

### 3.2. Effects of Salinity on Water-Related Parameters

**Figure 3:**
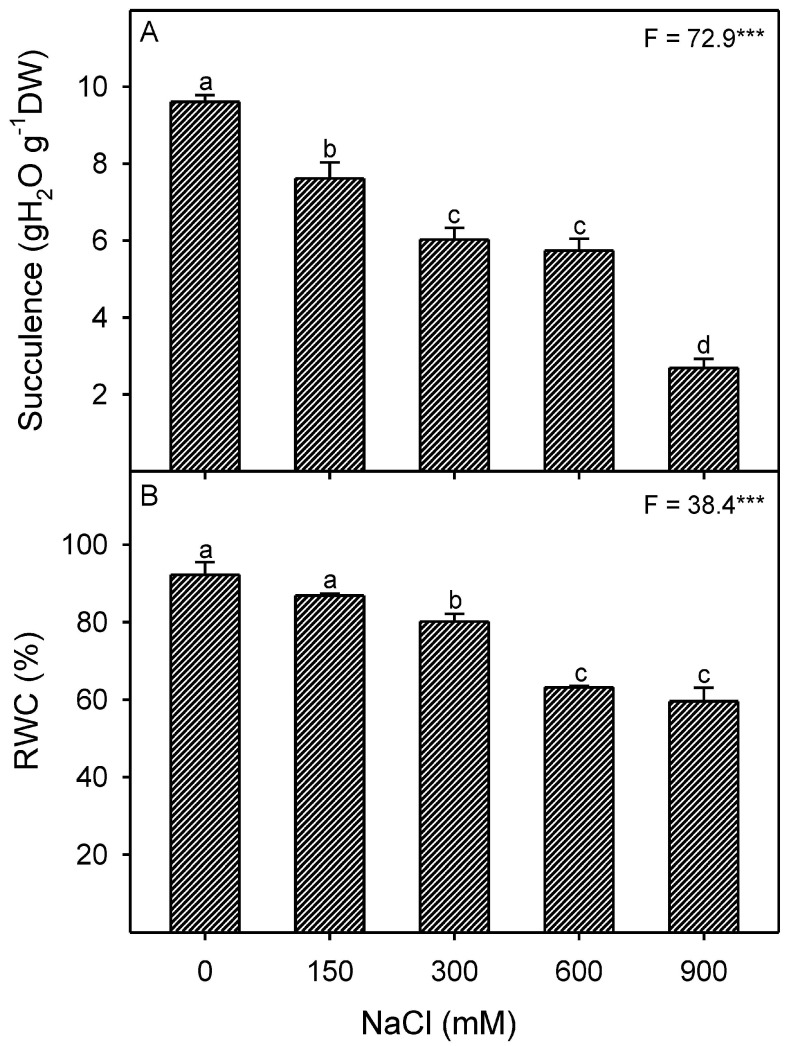
Effects of different NaCl treatments (0, 150, 300, 600, and 900 mM) on (**A**) succulence (g H_2_O g^−1^ DW) and (**B**) relative water content (RWC %) of *Zygophyllum propinquum* leaves. Bars represent mean ± standard error (n = 3). Bars with different letters are significantly different from each other (*p* < 0.05; post-hoc test). *F*-values based on one-way ANOVA for the effect of salinity are given. Where, *** = *p* < 0.001.

**Figure 4:**
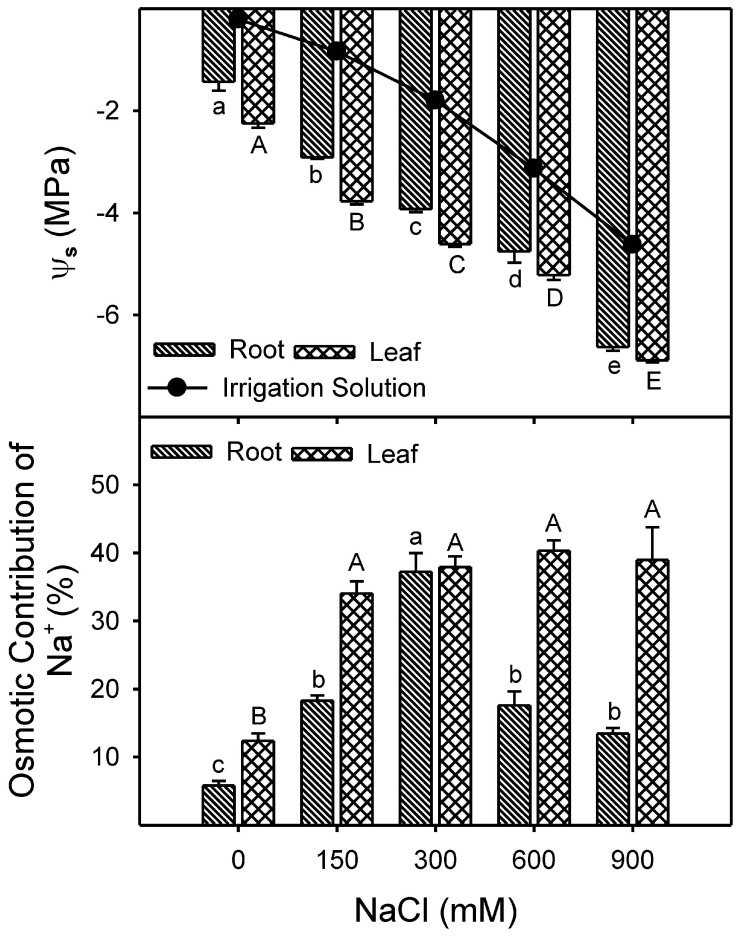
Effect of different NaCl treatments (0, 150, 300, 600, and 900 mM) on Osmotic potential *Ψ_s_* (MPa) and Percent osmotic contribution of Na^+^ in *Zygophyllum propinquum* roots and leaves. Bars represent mean ± standard error (n = 3). The circles represent the osmotic potential of the irrigation solution. Bars with different letters are significantly different from each other (*p* < 0.05; post-hoc test).

Shoot succulence decreased under saline conditions, while root water content also showed a reduction (Table 1). Leaf succulence and relative water content (RWC) decreased with increasing NaCl concentration (Figure 3A,B). However, root and leaf osmotic potential decreased (became more negative) progressively (*p* < 0.001) with increasing NaCl treatments, with generally lower values in leaves under high salinity (Figure 4). Percent osmotic contribution of Na+ in root and leaf samples were also affected by salinity (Figure 4).

### 3.3. Effect of Salinity on the Concentration, Content, and Tissue-Specific Distribution of Na^+^ and K^+^

**Figure 5:**
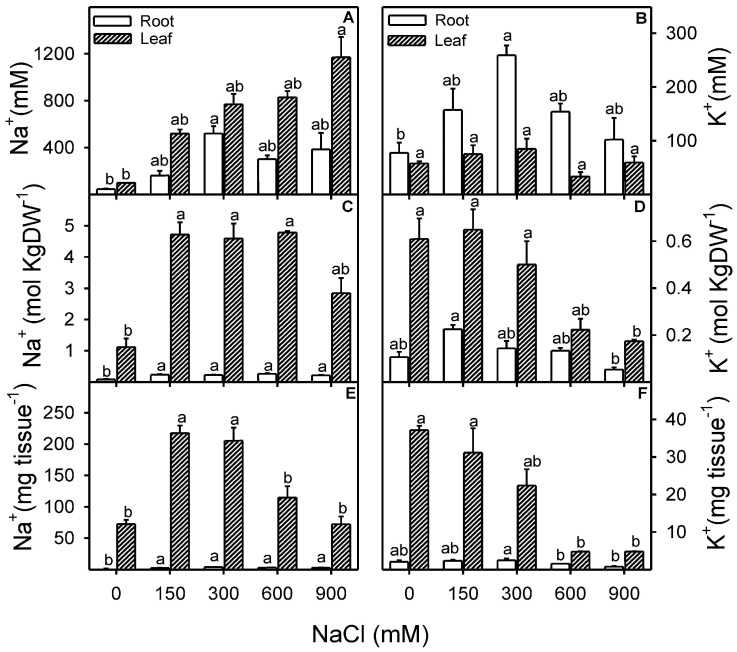
Effects of various levels of NaCl treatments (mM) on (**A**) Na^+^ concentration in root and leaf; (**B**) K^+^ concentration in root and leaf; (**C**,**E**) Na^+^ content in root and leaf; (**D**,**F**) K^+^ content in roots and leaves of *Zygophyllum propinquum*. Bars represent the mean ± standard error. Bars with different letters are significantly different from each other (*p* < 0.05; Bonferroni test).

An increase in salinity significantly affected ion flux in both the tissues (leaf and root) of *Z. propinquum*. Plants showed a linear increase in Na^+^ concentration with increasing NaCl dose, and this effect was more pronounced in leaf tissue (Figure 5A). K^+^ (mM) in the leaf remained unchanged in all NaCl treatments and the control, while K^+^ concentration in roots was substantially higher in the 300 mM NaCl treatment than in other NaCl and control treatments (Figure 5B). Leaf and root samples in all NaCl treatments showed significant (Root; F = 11.5, *p* < 0.01; Leaf; F = 13.4, *p* < 0.01) increases in Na^+^ content compared to the control. However, leaf tissue had > 10 folds Na^+^ than root tissue up to 300 mM NaCl. Meanwhile, root K+ content was unaffected by NaCl treatment, except when increased at 150 mM NaCl. Leaf K^+^ content was threefold higher than root K^+^ in salinity up to 300 mM NaCl treatment, while this difference was compromised with further increment in salinity dose. The Na^+^ and K^+^ ion distribution change based on the plant tissue biomass followed a similar trend to the ion content, except for an earlier decline in Na^+^ (from 600 mM NaCl) and K^+^ (from 300 mM NaCl) content in leaf tissue.

**Figure 6:**
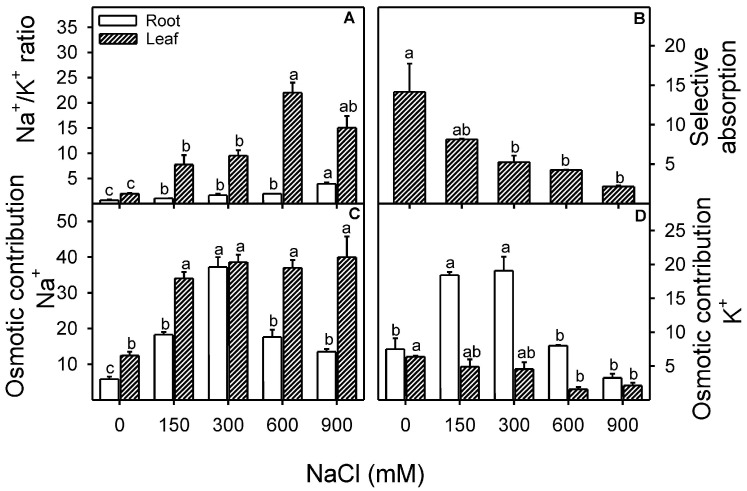
Effects of various levels of NaCl treatments (mM) on (**A**) Na^+^ to K^+^ ratio in root and leaf; (**B**) K^+^ selective absorption over Na^+^ (**C**) osmotic contribution of Na^+^ in root and leaf; (**D**) osmotic contribution of K^+^ in roots and leaves of Zygophyllum propinquum. Bars represent mean ± standard error. Bars with different alphabets are significantly different from each other (*p* < 0.05; Bonferroni test).

Na^+^ to K^+^ ratio significantly (Root; F = 49.7, *p* < 0.001; Leaf; F = 16.7, *p* < 0.001) increased with an increase in NaCl treatment, although this trend was more prominent in the case of leaf tissue (Figure 6A). Selective absorption of Na^+^ over K^+^ showed a significant (F = 8.3, *p* < 0.001) decline with an increase in NaCl treatment compared to the control (Figure 6B). The contribution of Na^+^ to leaf osmotic potential increased significantly (Leaf; F = 20.9, *p* < 0.001), and the maximum was found to be around 40% at 900 mM NaCl. However, Na^+^ contribution to root osmotic potential was around 20% in all NaCl treatments except 40% in 300 mM NaCl (Figure 6C). In contrast to Na^+^, the contribution of K^+^ to root osmotic potential declined with increasing NaCl treatment, while K^+^ contribution to shoot osmotic potential was ~20% up to 300 mM NaCl and less than 10% at ≥600 mM NaCl.

### 3.4. Effects of Salinity on Photosynthetic Pigments

Photosynthetic pigments (chlorophyll a, b) increased progressively from 150 to 600 mM NaCl and, although slightly reduced at very high salt (900 mM NaCl) relative to lower salt treatments, remained higher than in the control. Carotenoids remained unchanged; similarly, plants maintained comparable chlorophyll a/b and chlorophyll/carotenoid ratios across all NaCl treatments (Table 2).

### 3.5. Effects of Salinity on Chlorophyll Fluorescence Parameters

**Figure 7:**
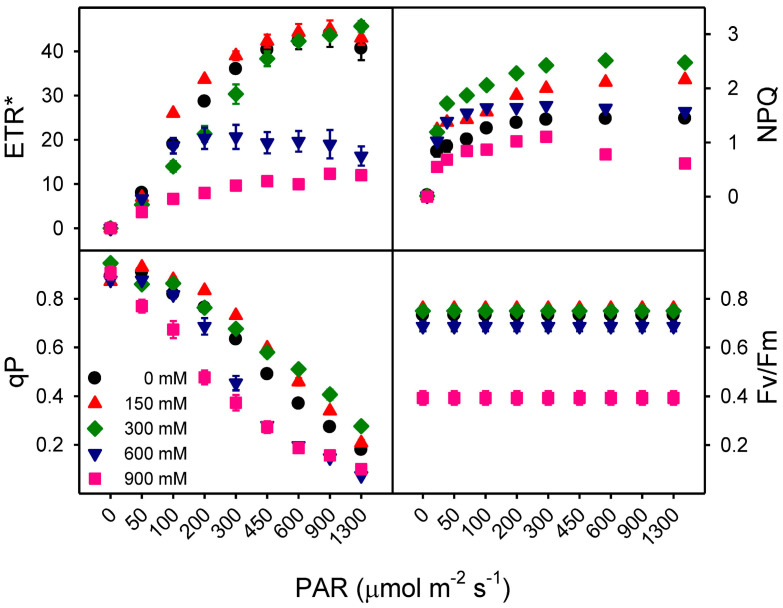
The effects of different NaCl treatments (0, 150, 300, 600, and 900 mM) and range of irradiance on the light response curve of *Zygophyllum propinquum* indicating absolute electron transport rate (ETR*), non-photochemical quenching (*NPQ*), photochemical quenching (*qP*), and maximum quantum efficiency (*F_v_*/*F_m_*). Each symbol represents the mean values ± standard error of five replicates.

Maximum efficiency of PSII (Fv/Fm) was unaffected at 150 and 300 mM NaCl (Fv/Fm: ~0.74), showed slight photo-inhibition at 600 mM NaCl (from ~0.74 to 0.69) and strong photo-inhibition (Fv/Fm: 0.39) at 900 mM NaCl (Figure 7). The absolute electron transport rate (ETR*) values increased with increasing irradiance. The ETR* remained unchanged (between 44 and 45) among low to moderate NaCl treatments (150 and 300 mM) and control plants; however, higher salinities (600 and 900 mM) reduced ETR* (Figure 6A). A reduction in photochemical quenching (qP) with increasing light intensity was observed in all salinity treatments. However, this decrease was more pronounced in the high-salinity treatments (600 and 900 mM) (Figure 7). Non-photochemical quenching (NPQ) increased at low to moderate salinity (150 and 300 mM) with increasing irradiance, but there was no significant change in non-photochemical quenching (NPQ) at the highest (900 mM) NaCl treatment with increasing irradiance (Figure 7). The recorded values in the light response curve of alpha (electron produced per photon) were lowest at 900 mM NaCl with a gradual decrease in other NaCl treatments, while the maximal rate of electron transfer ETRmax significantly decreased at high salt concentrations while remaining unchanged in 0, 150, and 300 mM NaCl. Similar trends were observed in the Ik value, with a slight increase at 300 mM NaCl (Table 3).

**Figure 8:**
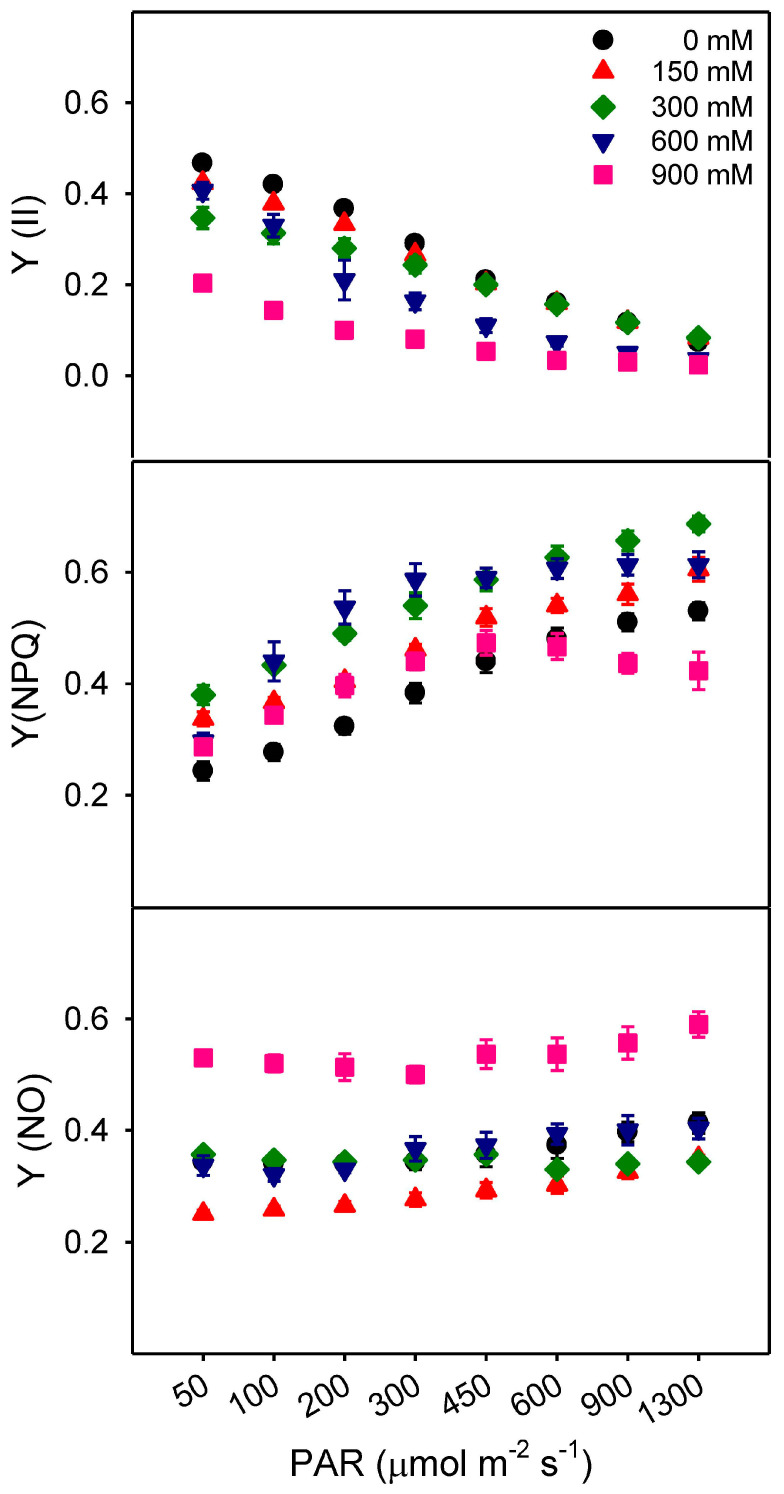
The effects of different NaCl treatments (0, 150, 300, 600, and 900 mM) and range of irradiance on the light response curve of *Zygophyllum propinquum* indicating relative fluorescence yields Y(II), non-photochemical quenching (YNPQ) and non-regulated photochemical quenching (YNO). Values are the mean of five replicates with mean ± standard error.

The results show that under increasing NaCl treatments with increasing irradiance, there was a rapid drop in Y(II), particularly under 900 mM NaCl at low irradiance. There is a corresponding rise in the yield of non-photochemical quenching (YNPQ), particularly under 300 mM NaCl, and a dip under 900 mM NaCl with increasing irradiance. The Y(II) values did not change with increasing NaCl concentrations but significantly decreased with increasing irradiance (Figure 8). The YNPQ values were slightly higher in the NaCl treatment compared to the control, but they were significantly lower in the highest salinity treatment (900 mM NaCl; Figure 8). Values of YNO were significantly higher in the 900 mM NaCl treatment compared to non-control saline and other NaCl treatments (Figure 8).

### 3.6. Effects of Salinity on Electrolyte Leakage

**Figure 9:**
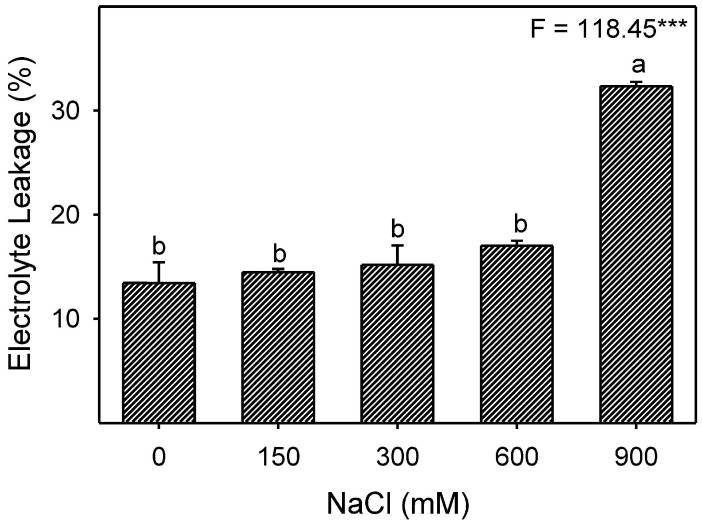
Effects of different NaCl treatments (0, 150, 300, 600, and 900 mM) on electrolyte leakage in *Zygophyllum propinquum* leaves. Bars represent mean ± standard error (n = 3). Bars with different alphabets are significantly different from each other (*p* < 0.05; post-hoc test). *F*-values based on one-way ANOVA for the effect of salinity are given. Where, *** = *p* < 0.001.

Low to moderate (150–600 mM NaCl) salinity did not cause any significant electrolyte leakage (EL); however, a substantially higher EL was observed at 900 mM NaCl (Figure 9).

### 3.7. Malondialdehyde (MDA) and Hydrogen Peroxide (H_2_O_2_) Contents

**Figure 10:**
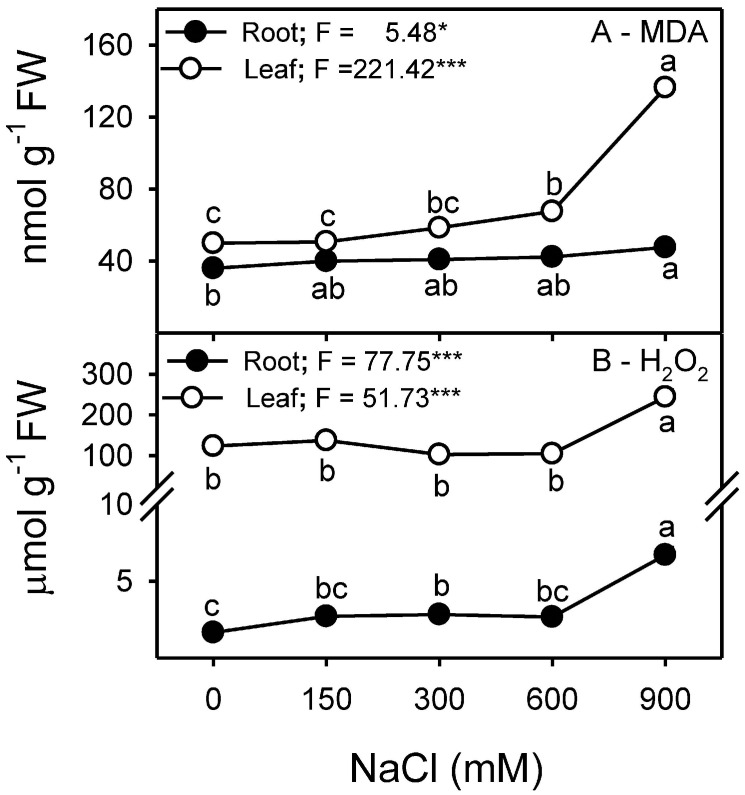
Effects of different NaCl treatments (0, 150, 300, 600, and 900 mM) on (**A**) MDA (nmol g^−1^ FW) and (**B**) H_2_O_2_ (µmol g^−1^ FW) content in the roots and leaves of *Zygophyllum propinquum*. Symbols represent the mean ± standard error (n = 3). Symbols with different letters are significantly different from each other (*p* < 0.05; post-hoc test). *F*-values based on one-way ANOVA for the effect of salinity are given. Where, * = *p* < 0.05 and *** = *p* < 0.001.

Contents of MDA, an indicator of lipid peroxidation, in roots were unaffected in response to salinity increments; however, a slight increase was observed at 900 mM NaCl. Likewise, a prominent increase in the MDA content of leaves occurred at 900 mM NaCl compared to other NaCl treatments (Figure 10A). H_2_O_2_ content of both roots and leaves increased substantially at 900 mM NaCl in comparison to other salt concentrations, with generally higher levels in leaves than in roots (Figure 10B).

### 3.8. Antioxidant Enzyme Activities

**Figure 11:**
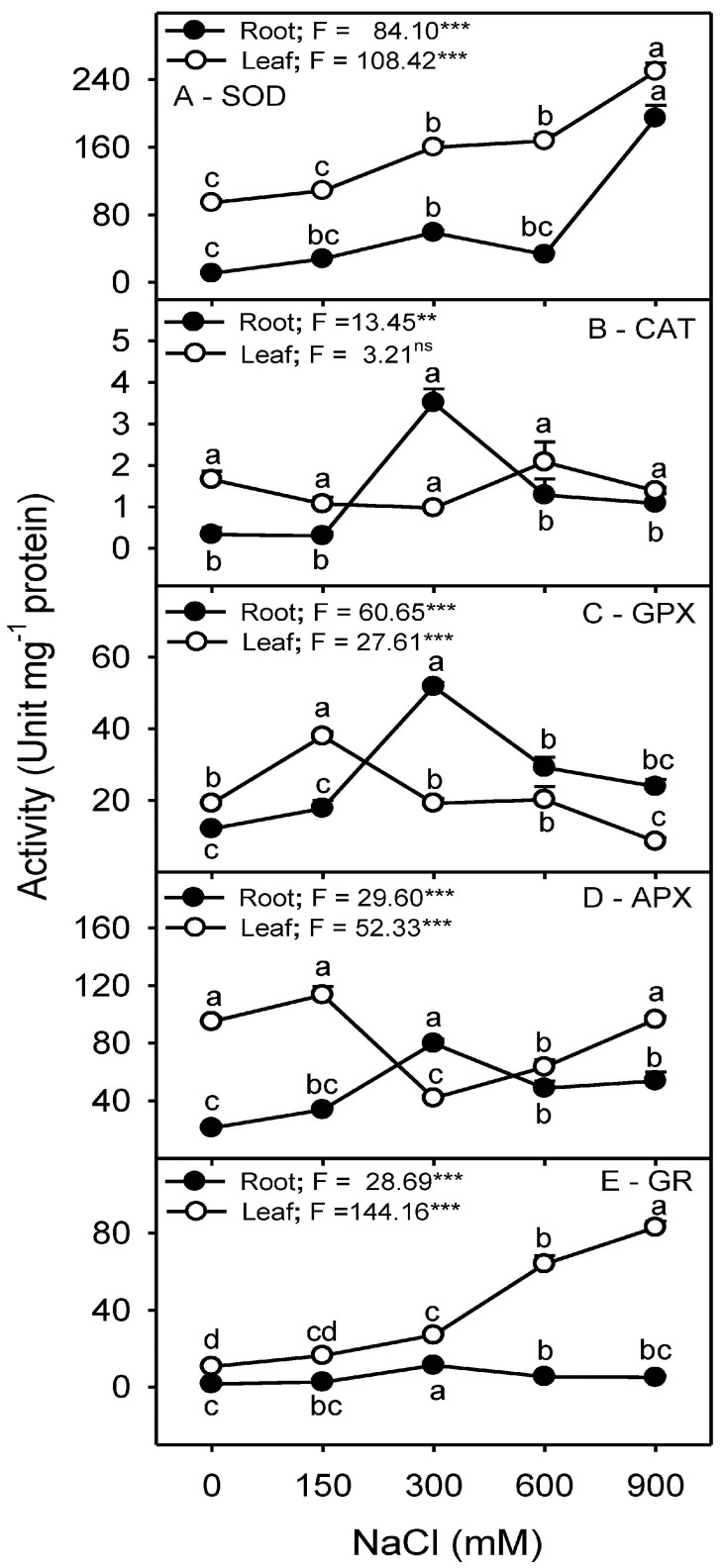
Effects of different NaCl treatments (0, 150, 300, 600, and 900 mM) on activity (Unit mg^−1^ protein) of (**A**) superoxide dismutase (*SOD*), (**B**) catalase (*CAT*), (**C**) guaiacol peroxidase (*GPX*), (**D**) ascorbate peroxidase (*APX*) and (**E**) glutathione reductase (*GR*) in roots and leaves of *Zygophyllum propinquum*. Symbols represent mean ± standard error (n = 3). Symbols with different alphabets are significantly different from each other (*p* < 0.05; post-hoc test). *F*-values based on one-way ANOVA for the effect of salinity are given. Where, ns = nonsignificant, ** = *p* < 0.01 and *** = *p* < 0.001.

Salinity had a significant (*p* < 0.001) effect on the activities of all antioxidant enzymes, except the CAT activity in leaves, which remained unchanged. Activities of CAT, APX, and GPX showed a similar trend in roots, with a transient increase at 300 mM NaCl (Figure 11). Whereas, in the case of leaves, the activity of CAT remained unchanged, and that of APX and GPX increased only transiently at 150 mM NaCl compared to other salinity treatments. Activities of SOD and GR in both root and leaf samples increased with increasing salinity (Figure 11).

### 3.9. Antioxidant Substrates

**Figure 12:**
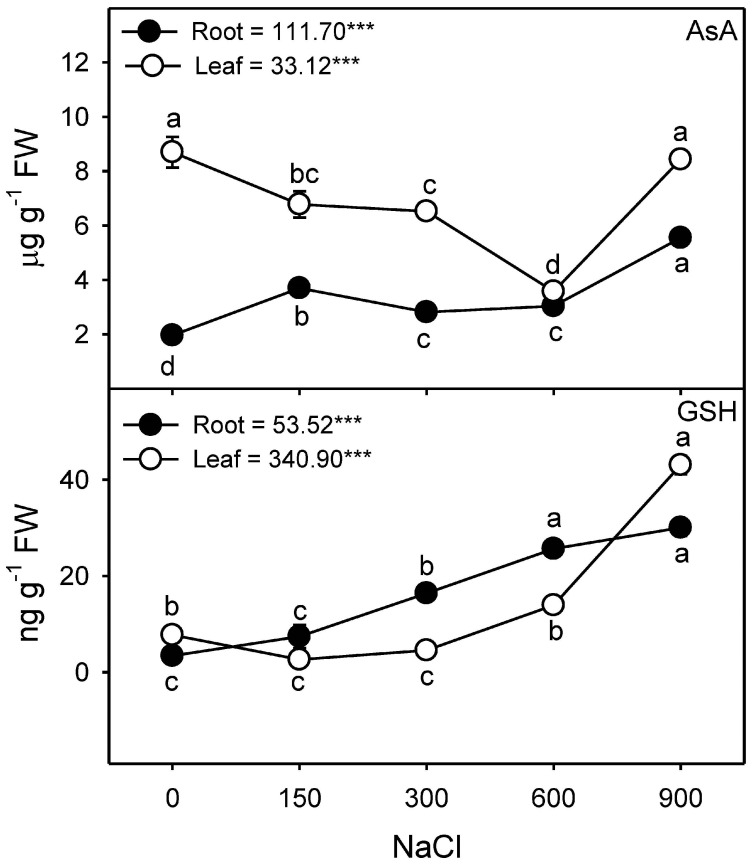
Effects of various NaCl treatments (0, 150, 300, 600, and 900 mM) on the contents of ascorbate (*AsA*) and glutathione (*GSH*) in root and leaf of *Zygophyllum propinquum*. Symbols represent mean ± standard error (n = 3). Symbols with different alphabets are significantly different from each other (*p* < 0.05; post-hoc test). *F*-values based on one-way ANOVA for the effect of salinity are given. Where, *** = *p* < 0.001.

Leaf AsA content decreased under low to moderate (150–600 mM NaCl) salinity and was comparable to control at the highest (900 mM NaCl) salinity (Figure 12). Whereas, root AsA levels were higher in salt-treated plants in comparison to non-saline control plants (Figure 12). Leaf GSH content did not change up to 300 mM NaCl but increased with increasing salinity; root GSH increased with increasing salinity (Figure 12).

## 4. Discussion

This study was planned to investigate the salt stress-related physio-chemical changes in *Z. propinquum.* Salinity stress negatively affects the growth and development of plants by retarding a number of physiological and biochemical metabolic functions [18]. The deleterious effects of salinity on plant growth include restricted water absorption and ion toxicity, leading to metabolic disturbances [100,101]. However, halophytes such as *Plantago coronopus* [15], *Zygophyllum xanthoxylum* [21,32], *Suaeda fruticosa* [102], *Salicornia dolichostachya* [103], *Limoniastrum guyonianum* [104], and *Salvadora persica* [105] displayed optimal growth and excellent adjustment to salt stress at low to moderate salt concentrations. In this study, optimal growth of *Zygophyllum propinquum* was observed in the non-saline control, which decreased with increasing salinity. However, leaf area reduced progressively with increasing salinity, and high leaf senescence at 900 mM NaCl could be termed as an adaptation to remove excess salts to survive under extreme saline conditions [106].

Changes in leaf-water relations are the prime indicators of stress among salt-treated plants [107] and may lead to ionic imbalances and metabolic changes [108,109]. Halophytes sequester ions such as Na^+^, Cl^−^, and K^+^ in their vacuoles to sustain osmotic balance under saline conditions and further counterbalance by synthesizing various organic solutes in the cytosol [21,27,28,110,111]. Xero-halophytes such as *Atriplex halimus* [29,30], *Atriplex canescens* [34], *Zygophyllum xanthoxylum* [21,33,101], *Sesuvium portulacastrum* [31], *Suaeda salsa* and *Kochia scoparia* [112] and *Nitraria tangutorum* [25] accumulate Na^+^ to resist water stress caused by high salt concentration in the growth medium. Similarly, many eu-halophytes are also reported to accumulate Na^+^ under salt stress *Sueada fruticosa* [102], *Gypsophila oblanceolata* [65], and *Salicornia dolichostachya* [103], which might help them in osmotic adjustment to achieve water balance. In the case of *Z. propinquum*, a linear decrease in osmotic potential (*Ψs*) and an increase in Na^+^ concentration in leaf tissues were observed, which is indicative of high Na^+^ accumulation in the shoot. However, decreased *Ψs* were more evident in leaf samples than in root samples, as observed in *Atriplex nummularia* [113]. Higher transport of Na^+^ from root to shoot and data on Na contribution in Leaf OP indicated that Na^+^ is used as a cheap osmotica for osmotic adjustment. Data indicates that the K+ content of the leaf was unchanged in the salt treatments. However, a significant reduction of K^+^ as compared to Na^+^ was observed in leaves under high salinity (up to 10 folds increased in leaf Na/K), as reported in many other halophytes such as *Arthrocnemum macrostachyum* [114], *Suaeda salsa, Kochia scoparia* [112], *Nitraria tangutorum* [25], and *Salicornia herbacea* [115]. An increased osmotic contribution of Na^+^ up to 300 mM NaCl represents better osmotic adjustment in root tissue. However, decreased Na^+^ and K^+^ contribution in roots with a further increase in salinity indicates that the plant is facing osmotic stress, which is also reflected in the case of compromised tissue water status. The decreased leaf succulence in *Z. propinquum*, with a parallel reduction in leaf osmotic potentials (*Ψs*), confirms that the plant’s lower growth is due to water stress.

The >20 folds higher Na^+^ content in leaf than root tissue from 150 mM NaCl (without any damage symptoms) represents the excellent salt accumulating strategy in aboveground tissues of *Z. propinquum*, as previously reported in many other halophytes like *S. fruticosa* and *Arthrocnemum macrostachyum* [102]. Whereas, the decline in Na content in shoots under high salinity (900 mM NaCl) is probably due to limited transpiration pull for Na uptake (as leaf area is severely reduced) and inhibition of the growth of aboveground biomass [106]. The high Na^+^ concentration in root tissue and the increasing trend of Na + concentration in shoots with salinity are due to lower tissue water content. *Z. propinquum* can maintain K concentration up to 900 mM NaCl (by lowering water content) for better performing all metabolic functions. However, the severely declined K content in the leaf is concomitant with the compromised ability of K over Na selective absorption and is probably due to high Na transport toward the shoot.

Photosynthetic pigments are usually analyzed to assess the effects of salinity on plant energy metabolism [116]. In general, photosynthetic pigments decrease under saline conditions in the halophytes *Sarcocornia fruticosa*, *Arthrocnemum macrostachyum*, *Salicornia europaea*, *Suaeda salsa*, *Kochia scoparia*, *Panicum turgidum*, and *Quinoa* [55,112,114,117,118,119]. However, in *Z. propinquum*, chlorophyll and carotenoid contents appeared to be generally unaffected by salinity treatments, with some increases in chlorophyll content at low to moderate NaCl treatments (150–600 mM) as in *Bruguiera parviflora* [120], *Chenopodium album* [121], *Atriplex portulacoides* [122], *Nitraria roborowskii* [123], and *Salvadora persica* [105]. An increase in chlorophyll content could be a function of reduced leaf succulence and a compensatory pigment turnover response under saline conditions. However, the chl a/b ratio (i.e., an indicator of antenna size, [124] and content of carotenoids (i.e., a protective pigment) remained unchanged across the treatments in this study, but the chl/car ratio increased in 600 mM NaCl. The increased chl content and chl/car with maintained chl a/b and Car content coincided with increased ETR by the support of high NPQ to improve NADP recovery to prevent photo-inhibition and protect photosystem II, which reflects the plant tolerance strategy up to 300 mM NaCl. In contrast, plants exposed to 600 mM NaCl showed a similar photo-pigment trend to low salinity treated plants but partially decreased ETR with some increase in NPQ [Y(NPQ)] to prevent photo-inhibition and protect photosystem II and shows the resistance strategy. This means that salt stress perhaps had no influence on the structure of the photosystems in the thylakoids in the tested species. As the half-life of the reaction center of PSII is in the range of 30 to 15 min (depending on low to high light intensity), this implies that the ratio of the rates of degradation versus de novo synthesis of protein complexes is also not affected by salt stress. Another succulent dicot halophyte, *Suaeda altissima*, retained its chloroplast ultrastructure and photosynthetic function in an intact condition in up to 750 mM NaCl [125]. Salinity (400 mM NaCl) led to an increase in PsaA/B, CP47, CP43, and Lhcb1 proteins with a concurrent increase in antennae size in the succulent halophyte *Salicornia bigelovii* [126]. In contrast, *Z. propinquum* plants suffered severe damage at 900 mM NaCl, as ETR decreased due to photo-inhibition (decreased Fv/Fm) and concomitant high Y(NO) alongside car content, compared to control plants. Hence, it appears that the test species maintained the chl a/b ratio (antenna size) with unaltered carotenoids across different salinity treatments (particularly up to 600 mM NaCl) to maintain its light-harvesting ability, as reported for another tolerant halophyte, *Salvadora persica* [105].

Chlorophyll fluorescence measurements, such as the maximum quantum yield of PSII (Fv/Fm), provide efficient insight into photo-inhibition at the biochemical level before actual symptoms of stress become visible [37]. In *Z. propinquum*, the Fv/Fm ratios were unaffected at low to moderate salt concentrations, similar to *Lycium barbarum* [127], *Sarcocornia fruticosa* [117], and *Spartina* sp. [128], *Thellungiella halophila* [129], with a sharp decline in the very high salt treatment (900 mM). Reduction in Fv/Fm from 0.74 in non-saline control to 0.38 under high salinity (900 mM) indicates chronic photo-inhibition and damage to chloroplast proteins due to failure of protective mechanisms [124]. Similarly, alpha (α) and ETRmax values were also the lowest at 900 mM NaCl, suggesting that plants were unable to sustain PSII performance, electron production ratio, and electron transport rates. The upregulation of NPQ (with an increase in the xanthophyll cycle) occurs due to a decreased PSII efficiency and high [H^+^] in the lumen. In *Z. propinquum*, the lowest NPQ values in 900 mM NaCl-treated plants indicate compromised defense (xanthophyll cycle) at the level of PSII. The Y(II) values, indicating linear electron flow through the electron transport chain, decreased sharply with increased NaCl treatments. This decrease was more prominent at low light intensities, which is typical of C_4_ plants [124]. The YNPQ values, which reflect enzymatically regulated non-photochemical quenching by heat dissipation, were higher under saline conditions, except for 900 mM NaCl. These results indicate that (YNO) is significantly contributes to excitation quenching by non-regulated photochemical energy loss at 900 mM NaCl for dissipation of excess light energy, which may also explain the sharp drop in Fv/Fm values. The sharp drop in Fv/Fm leads to ROS production as a result of photo-inhibition in the chloroplast, which is generally quenched by antioxidant enzymes [130].

The amount of membrane damage induced by stress is evaluated by determining solute or electrolyte leakage [131]. Electrolyte leakage is linked to a series of reactions related to the formation of free radicals [132], which causes membrane damage or lipid peroxidation [133] and is usually determined by the increased amount of MDA contents. No significant induction of electrolyte leakage or membrane peroxidation (as indicated by MDA) was observed in the leaves and roots of *Z. propinquity* in up to 600 mM NaCl. However, a drastic increase in both parameters was observed in the leaves at 900 mM NaCl. Similar results have been reported for *Sesuvium portulacastrum* [134], *Suaeda salsa* [135], *Tamarix chinensis* [136], and *Limonium bicolor* [137]. Levels of H_2_O_2_ (a common ROS) in the leaves and roots of *Z. propinquum* increased only at 900 mM NaCl, similar to the findings found in the roots of wild tomato plants [138] and *Crithmum maritimum* [139] or in the leaves of *Arabidopsis thaliana* [140], *Limonium stocksii* [69], and many other species [16,63,68,141]. Halophytes utilize a number of enzymatic and non-enzymatic antioxidants to quench ROS produced under salinity stress [68,102]. SOD activity increased progressively in response to salt stress in both root and leaf samples, as has already been reported in the roots and leaves of *Crithmum maritimum* [139], *Atriplex portulacoides* [122], and *Quinoa* [119]. In *Z. propinquum*, CAT activity remained unchanged in the leaves in all treatments; similarly, many reports have suggested unchanged or decreased levels of CAT under salinity [112,120,122]. Roots of *Z. propinquum* showed a transient increase in activities of CAT, APX, GPX, and GR at 300 mM NaCl, similar to the transient change in CAT and GR activities in roots of *Crithmum maritimum* plant [139]. The activity of GPX progressively decreased with an increase in salinity, as reported in *Salvadora persica* [105]. In contrast, the activity of APX of *Z. propinquum* increased at low NaCl concentrations and gradually decreased at high salt concentrations. Such a decrease in APX has also been reported in other halophytes [105,139]. Glutathione reductase (GR) activity in *Z. propinquum* was higher at high salt treatments (600 and 900 mM NaCl). The glutathione reductase activity was also stimulated by salinity in *Atriplex portulacoides* [122]. The higher activity of SOD, along with low APX, GR, and GPX activities, could be responsible for the higher H_2_O_2_ and MDA contents in *Z. propinquum* in the high-salinity treatment. Plants require AsA and GSH to maintain the photosynthetic machinery’s membrane integrity and as key non-enzymatic antioxidants [142,143]. Levels of Leaf AsA and GSH of *Z. propinquum* increased at high salinity; however, it was not enough to prevent damage at 900 mM NaCl.

## 5. Conclusions

**Figure 13:**
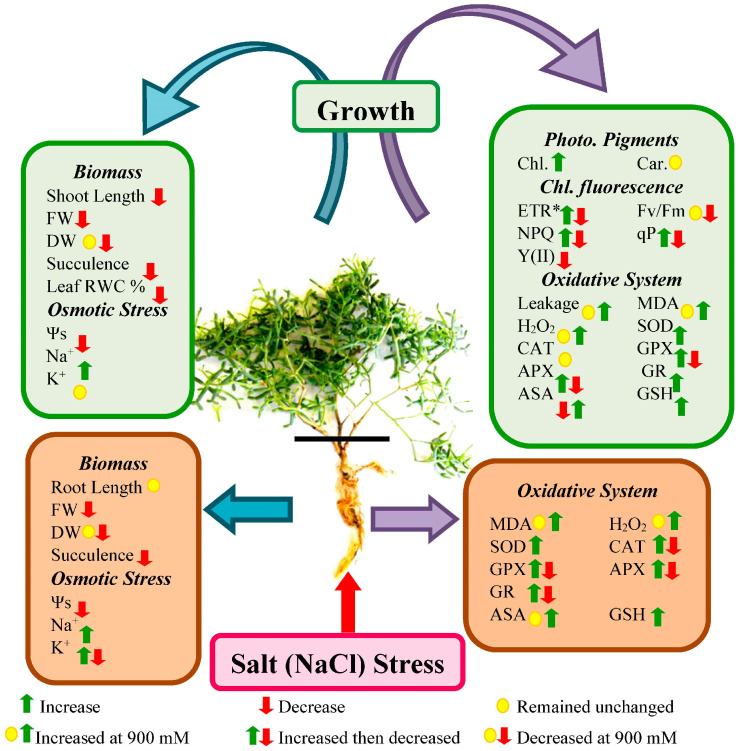
Summary of the physiological and biochemical responses of *Zygophyllum propinquum* during vegetative growth under various NaCl treatments (0, 150, 300, 600, and 900 mM). Green boxes represent leaf parameters and brown boxes represent roots.

The results of this study indicate that *Z. propinquum* modulates both growth and physicochemical parameters to cope with increasing salinity (Figure 13). A decrease in fresh biomass and succulence was accompanied by higher tissue Na^+^, which probably caused a decrease in the sap osmotic potential upon exposure to salinity. Generally, chlorophyll fluorescence parameters remained unaffected in up to 600 mM NaCl concentrations. However, high salt concentrations (900 mM) caused a reduction in Fv/Fm. Similarly, the oxidative damage parameters increased only at 900 mM NaCl, indicating significant damage by high salinity. Hence, growth reductions under low to moderate (150–600 mM NaCl) salinity appear to result from the energetic cost of salt resistance, while high (900 mM NaCl) salinity was injurious.

## Figures and Tables

**Table 1:** Effects of different NaCl treatments (0, 150, 300, 600, and 900 mM) on root and shoot length, fresh weight (FW), dry weight (DW), and succulence (Suc.) of *Zygophyllum propinquum* plant. Values represent mean ± standard error (n = 3), and different letters indicate that values are significantly different from each other (*p* < 0.05; post-hoc test). F-values based on a one-way ANOVA for the effect of salinity are provided. Where, ns = non significant, ** = *p* < 0.01 and *** = *p* < 0.001.

Tissue	Parameters	0	150	300	600	900	F-Value
		mM (NaCl)					
Root	Length (cm)	14.56 ± 0.74 a	15.58 ± 1.25 a	15.16 ± 0.99 a	13.29 ± 0.25 a	12.55 ± 1.17 a	1.78 ^ns^
	FW (g plant^−1^)	1.88 ± 0.09 a	1.18 ± 0.11 b	1.11 ± 0.06 b	1.03 ± 0.04 b	1.01 ± 0.05 b	23.38 ***
	DW (g plant^−1^)	0.69 ± 0.02 a	0.62 ± 0.04 a	0.77 ± 0.04 a	0.56 ± 0.04 ab	0.46 ± 0.04 b	7.82 **
	Suc. (gH_2_Og^−1^DW)	1.73 ± 0.05 a	1.84 ± 0.08 a	0.44 ± 0.08 c	0.85 ± 0.01 b	0.64 ± 0.12 bc	22.22 ***
Shoot	Length (cm)	22.86 ± 0.82 a	21.33 ± 0.44 a	20.74 ± 0.99 a	17.86 ± 0.93 b	16.59 ± 0.75 b	10.08 **
	FW (g plant^−1^)	36.82 ± 1.33 a	27.75 ± 0.22 b	19.09 ± 0.88 c	14.38 ± 0.74 d	8.69 ± 0.33 e	188.90 ***
	DW (g plant^−1^)	3.66 ± 0.38 a	3.90 ± 0.25 a	3.45 ± 0.04 a	2.93 ± 0.06 b	2.86 ± 0.09 b	12.95 **
	Suc. (gH_2_Og^−1^DW)	9.21 ± 0.68 a	6.18 ± 0.44 b	4.53 ± 0.25 b	4.06 ± 0.14 c	2.04 ± 0.18 c	34.18 ***

**Table 2:** Effects of different NaCl treatments (0, 150, 300, 600, and 900 mM) on photosynthetic pigments of *Zygophyllum propinquum* leaves. Values represent mean ± standard error (n = 3), and different letters show that values are significantly different from each other (*p* < 0.05; post-hoc test). F-values based on one-way ANOVA for the effect of salinity are given. Where, ns = nonsignificant, * = *p* < 0.05, ** = *p* < 0.01 and *** = *p* < 0.001.

Parmeters	0	150	300	600	900	F-Value
	NaCl (mM)					
Chlorophyll a	0.63 ± 0.02 b	1.31 ± 0.07 a	0.98 ± 0.06 ab	1.19 ± 0.14 a	0.87 ± 0.01 ab	13.42 **
Chlorophyll b	0.43 ± 0.01 b	0.69 ± 0.01 ab	0.85 ± 0.12 a	0.82 ± 0.08 a	0.48 ± 0.01 b	8.96 *
Total chlorophyll	1.06 ± 0.02 b	2.01 ± 0.06 a	1.83 ± 0.12 a	2.00 ± 0.12 a	1.35 ± 0.01 b	27.84 ***
Carotenoids	0.16 ± 0.00 a	0.17 ± 0.02 a	0.22 ± 0.02 a	0.19 ± 0.02 a	0.20 ± 0.01 a	2.17 ^ns^
a/b ratio	1.47 ± 0.05 a	1.89 ± 0.10 a	1.21 ± 0.23 a	1.51 ± 0.26 a	1.81 ± 0.04 a	2.86 ^ns^
ch/car ratio	6.64 ± 0.25 c	11.86 ± 0.85 a	8.69 ± 1.60 b	11.48 ± 1.56 a	6.91 ± 0.15 c	5.20 **

**Table 3:** Effects of different NaCl treatments (0, 150, 300, 600, and 900 mM) on additional photosynthetic parameters of *Zygophyllum propinquum*. Values represent mean ± standard error (n = 5), and different letters show that values are significantly different from each other (*p* < 0.05; post-hoc test). F-values based on one-way ANOVA for the effect of salinity are given. Where, *** = *p* < 0.001.

Parameters	0	150	300	600	900	F-Value
	NaCl (mM)					
ETR_max_	43.33 ± 2.20 a	44.95 ± 1.25 a	47.07 ± 1.09 a	16.43 ± 1.35 b	13.23 ± 0.61 b	140.43 ***
F_v_/F_m_×ETR/2	0.31 ± 0.00 b	0.32 ± 0.00 a	0.32 ± 0.00 b	0.30 ± 0.00 c	0.18 ± 0.00 c	481.46 ***
I_K_	184.07 ± 8.43 a	230.78 ± 7.27 a	170.30 ± 2.50 a	98.43 ± 0.86 a	91.83 ± 1.52 b	116.87 ***
Alpha	0.23 ± 0.00 a	0.21 ± 0.01 a	0.17 ± 0.00 b	0.17 ± 0.01 b	0.09 ± 0.00 c	114.12 ***

## Data Availability

The original contributions presented in the study are included in the article, further inquiries can be directed to the corresponding author.
